# GIBLIB (4.0)

**DOI:** 10.5195/jmla.2022.1466

**Published:** 2022-07-01

**Authors:** Heather Healy

**Affiliations:** 1 heather-healy@uiowa.edu, Clinical Education Librarian, Hardin Library for the Health Sciences, University of Iowa, Iowa City, IA.

## Abstract

**GIBLIB (4.0).** GIBLIB, 811 W 7Th Street, 13th Floor, Los Angeles, CA; https://www.giblib.com/; Contact for institutional pricing https://www.giblib.com/services/institutional-pricing; Individual pricing structure: Basic, Standard, and Premium plans. Compatible with PC, Mac, tablet, and mobile.

## PURPOSE AND GENERAL DESCRIPTION

GIBLIB, which sometimes refers to itself as the “Netflix of medical education,” [1] is an on-demand, streaming video resource providing medical education on a wide variety of topics in many disciplines. Videos include surgical procedures, lectures, expert advice, one-on-one talks, and courses (i.e., collections of videos on a topic).

Company founders are CEO Brian Conyer and co-founder Jihye Shin [1]. The chief medical officer is practicing emergency medicine physician Omeed Saghafi, MD, MBA [2]. The company aims to bring studio-quality content to medical education, and their mission is “to empower and inspire healthcare professionals everywhere to never stop learning” [3]. The GIB in the resource's name honors the inventor of the heart– lung machine, Dr. John Heysham Gibbon, and LIB is short for library [4].

## INTENDED AUDIENCE

The target audience for GIBLIB is healthcare providers, trainees, and students. The experts featured in the resource primarily include physicians, but advanced practice providers, physical therapists, pharmacists, and others in healthcare roles are represented. The coverage includes a wide variety of specialties, making the content relevant to a broad health sciences audience.

The videos can be used by practicing providers for their own learning and continuing education. Continuing Medical Education (CME) credit is available through the premium level individual subscription and may be an option for institutional subscribers. The content can be used by students and trainees to explore any topic or specialty of interest as well as a supplement to educational programs for these groups. Procedural videos (e.g., surgeries, physical exams) in particular can be used to supplement educational programs or to augment training in the instance of in-person learning disruptions such as those caused by the COVID-19 pandemic.

## USABILITY AND MAJOR FEATURES

Navigation in GIBLIB is easy and intuitive. Users can explore the content in several ways: search by keyword, browse by category, or view the catalog. Most aspects of the resource can be accessed seamlessly without registering for a personal account or logging in; institutional users do need to create an account and log in to make use of bookmarks, CME (if included), and playlists.

**Figure 1:**
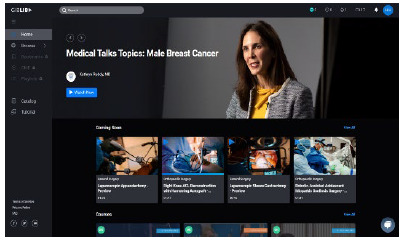
Screenshot of the GIBLIB main interface.

From the main interface, a left sidebar provides navigation to “Home,” “Browse,” “Bookmarks,” “CME,” “Playlists,” “Catalog,” and “Tutorial” (Figure 1). The Home link returns users to the main interface. Browse lets users choose to view content by “Specialties,” “Conferences,” “Courses,” “Organizations,” “Experts,” “Hot Topics,” and “360VR.” Several of these categories have more granular sublevels to allow users to choose more specific content. The “Bookmarks” page displays videos a user has marked. The “CME” page tracks completed credits (if available). Playlists provide the option for users to create their own collections of videos. Adding a video to any playlist is simple with a couple of clicks. Currently, no option exists for sharing playlists; however, such a feature would be helpful for instructors who would like to curate collections for and assign them to students and trainees. The “Catalog” provides a bird's eye view of the entire library's contents, allowing users to bookmark or add videos to playlists directly from this interface.

The resource is designed to work on desktop, tablet, or phone, and all features are available on all devices with Chrome being the recommended browser. On a smaller laptop screen, the long lists of “Specialties” subtopics in “Browse” could not be viewed at once. With touchscreen or arrow keys, moving the list to access the remaining subtopics was possible but not intuitive. Similarly, on the reviewer's iPhone, the more granular levels of the “Specialties” were difficult to select and access. Navigating the video carousels on the main page was difficult on a laptop screen but worked seam-lessly on phone and tablet. Perfecting the ease of access across devices would make the resource more user friendly for faculty and students who would be likely to access the resource from a wide variety of devices.

The video player controls are similar to those users are likely to have experienced on other streaming video platforms, including skip back 10 seconds, skip ahead 10 seconds, sharing options, playback rate, captions, picture-in-picture mode, full-screen mode, and playback quality levels. The captions are available in English, Spanish, Korean, and simplified Chinese, and they are powered by Google. As such, errors do occur and are similar to those seen in other software with automatic captioning or transcription features. The transcripts appear to be based on the captions, so errors may be found. However, the transcripts are fully searchable and allow users to skip ahead to a specific place in the video based on the text. Notes allow users to save any information learned from a video; notes created for a video can also be downloaded.

## CONTENT

As of this writing in January 2022, the resource contains nearly 4,500 videos. Of these, 75% of the videos are associated with Mayo Clinic; the remaining 25% of videos are affiliated with another 14 organizations, including Society for Thoracic Surgeons and Cleveland Clinic. New content is added regularly [4].

**Figure 2:**
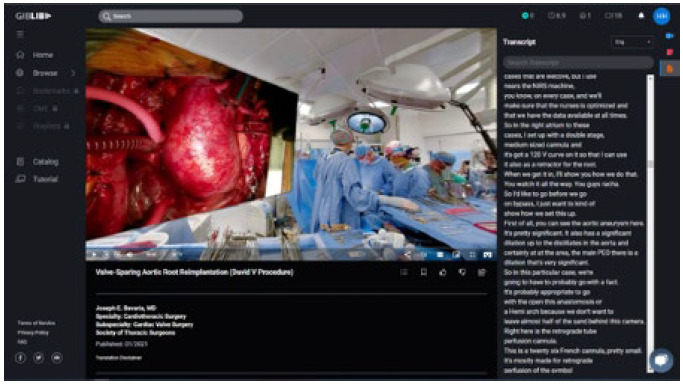
Screenshot of a 360VR surgery video with transcript displayed.

Videos come in a variety of types. Procedural videos are primarily surgeries and include general, plastic and reconstructive, cardiothoracic, orthopaedic, and oral and maxillofacial surgery. Some surgery videos are 360 virtual reality (360VR). In these videos, the user sees a wider shot of the operating room and simultaneously a virtual screen showing a close view of the procedure itself (Figure 2). Users can navigate with a mouse or touch to rotate their view 360 degrees to center on the closeup virtual screen or to see all around the operating room. Chrome is recommended when viewing the 360VR videos. For virtual reality headsets, users can use the native browser to access the 360VR collection [4]. Other procedural-type videos include those demonstrating physical exam skills, such as a concussion exam or telemedicine musculoskeletal exam.

Other video types are in the format of one-on-one conversations, interviews, conference presentations, discussions, or lectures, and cover a variety of topics. Some present a featured speaker talking about a particular area of expertise which may run anywhere from a few minutes to a halfhour or more. Many of the lecture and conference presentation videos provide the audio of the speaker and show a slide deck. On occasion a slide deck may have blanks, which occurs when content is removed due to copyright restrictions [4].

The metadata for each video provides a brief description of the content, the name of the expert, the affiliated institution, filming date, and publication date. A beneficial addition would be a short list of objectives accomplished by the video enabling medical educators to determine whether a particular video might be appropriate for an educational purpose. This would help educators determine whether to invest their time in watching the video prior to assigning it to trainees or students. Another feature not currently available would be an option for creating a citation for any video and then exporting the citation to a reference management software allowing students and faculty to incorporate the material into lectures or class assignments easily. Even a suggested format for citing any video in the FAQ section would be of use.

In the “Browse” section of the navigation sidebar, “Specialties” currently includes 36 content areas (“Internal Medicine,” “Pharmacology,” “Nurse Practitioner,” etc.) with many of these content areas including subspecialties. The amount of content for each specialty varies; for example, many more videos are available for “Surgery” and “Internal Medicine” than for “Dermatology” and “Emergency Medicine.” Users can also browse by “Conferences,” which contain a variety of videos, such as recordings of a COVID-19 Live Webinar Series from Mayo Clinic. However, this section appears to be in development because at the time of this writing some of the collections have no content assigned. Another browsing option is “Courses” which provides predefined collections of videos about a specific topic. Some examples include GIBLIB and Cedars Sinai Present: Topics in Gender Dysphoria (3 videos), Opioids (9 videos), or Care of the Older Adult (15 videos). Users can also browse by specific “Organizations,” “Experts,” or “Hot Topics.”

## PRICING LEVELS AND PURCHASE OPTIONS

Institutional pricing is customized according to the size of the organization. Institutions should contact GIBLIB for further details.

Free trials lasting 4 days are available for any user. Individual subscription plans include the basic plan at $20/month (or $200 if billed annually), the standard plan at $60/month (or $360 if billed annually), and the premium plan (includes CME) at $83/month for one year. The premium plan can also be purchased for 2 years ($1500 one-time payment) or 3 years ($1800 one-time payment). A discount of 50% is available on individual subscriptions for residents and students.

## CONCLUSION

Though many health sciences resources provide some video content, the exclusive focus on videos as well as the depth and breadth of topics available make GIBLIB a unique and excellent medical education resource for faculty, trainees, and students. The resource will meet many individual users own learning goals within their specialty but also provides them with options to explore topics outside of their specific discipline. The platform has great potential as a resource that faculty can use in supporting the curriculum of a number of health sciences educational disciplines. The ease of use on all devices will allow users to watch content on whatever platform they prefer, and the options for bookmarking and creating playlists allows users to prioritize, customize, and manage their learning.
